# Advantages of Short-Segment Fusion in the Surgical Management of Thoracolumbar Traumatic Fractures: A Case Series and Review of the Literature

**DOI:** 10.7759/cureus.39535

**Published:** 2023-05-26

**Authors:** Luke Mugge, Danielle D Dang, Omar Awan, Megan Vaughan, Wenli Mui, Cristie Brewer, Conner Dominick, John Hamilton

**Affiliations:** 1 Neurological Surgery, Inova Neuroscience and Spine Institute, Falls Church, USA; 2 Neurological Surgery, Inova Fairfax Medical Campus, Falls Church, USA; 3 Neurosurgery, Inova Neuroscience and Spine Institute, Falls Church, USA

**Keywords:** corpectomy, surgical management, thoracolumbar, trauma, short segment

## Abstract

Introduction: Spine trauma is a common pathology that frequently requires neurosurgical intervention. Few studies have examined short-segment, 360-degree stabilization of traumatic thoracolumbar fractures.

Methods: A retrospective review was completed of adult and pediatric patients who underwent surgical correction for thoracolumbar fractures between December 2011 and December 2021.

Results: Forty patients met the inclusion criteria. The majority of patients presented with an American Spinal Injury Association (ASIA) score of D (n = 11) or E (n = 21). The most common level of injury was L1 (n = 20). The average length of stay was 11.7 days. Postoperatively, two patients had pulmonary emboli or deep venous thrombosis, and two had surgical site infections. Most patients were discharged to home (n = 21) or acute rehab (n = 14). The fusion rate at six months was 97.5%. Neurologically, all patients regained ambulation by >18 months follow-up. For the ASIA scale, most had a score of D (n = 4) or E (n = 32) at six months. The same trend was observed with the Frankel score, where most patients had either D (n = 5) or E (n = 31), improving to only two having a score of D at >18 months.

Conclusions: Corpectomy followed by posterior fusion has a number of biomechanical benefits. This construct permits circumferential decompression, larger surface area for fusion, improved reconstitution of vertebral body height, reduced kyphosis, and an overall shorter segment. This results in fewer levels needing to be fused while enabling the greatest changes of successful fusion.

## Introduction

Spine-related trauma is a common pathology that represents a prominent neurosurgical concern, with an estimated prevalence of approximately 3% of all trauma-related injuries [1]. Demographically, there is an unequal representation of patients, as approximately two-thirds of spine-related traumas are in males, with injuries occurring between the ages of 20 and 40 years [2]. The etiology of most spinal cord injuries (SCIs) is high-velocity impact with sudden deceleration [3].

The thoracolumbar spine represents the most common site of injury. Injuries frequently include transition levels (T11-L2), where load transmission is the most impactful [2]. Of these injuries, 16% are fracture-dislocations, and given the nature of the initial injury, approximately half of the patients will have concomitant neurological deficits [4].

Several classification systems have been developed to guide operative and nonoperative management. The Denis three-column model of thoracolumbar spine stability is the most popular means of assessment, as it only requires a computed tomography (CT) and has relatively good predictive value [5]. There are three degrees of instability, with a grade 1 representing mechanical instability and increased acuity up to a grade 3, which is both mechanically unstable and involves neurological injury. In terms of deciding whether operative intervention is necessary, the thoracolumbar injury classification and severity (TLICS) score is commonly applied [6]. Being a simple classification, which consists of only three categories (morphology, integrity of the posterior longitudinal ligament, and neurological status), the TLICS score stands as a proven and validated method of assessing the need for fusion [6].

In terms of management, bracing versus surgical stabilization defines the two broad categories of management, with each pursued depending on the relative degree of mechanical spinal stability determined radiographically [7,8]. The patient's baseline neurological status presentation is also of utmost importance. Once surgical intervention is deemed necessary, selecting the appropriate approach is crucial. Despite being a common pathology, there are no consensus guidelines for the most appropriate surgical intervention for complex bursts or chance fractures occurring in a given patient with a given neurological status. We sought to examine our institutional experience treating thoracolumbar burst and chance fractures with a combination of anterior/posterior approaches, hypothesizing that having circumferential fixation would permit increased stability, require fewer involved levels, preserve the range of motion, and achieve primary stability.

## Materials and methods

Study design and patient population

Prior to the outset of the study, approval was obtained from the Institutional Review Board (Inova Health System Foundation IRB; U21-09-45357). A retrospective review was conducted of adult and pediatric patients who had undergone surgical correction for thoracolumbar burst or chance fractures between December 2011 and December 2021, spanning a full 10-year period. Patients were reviewed for inclusion after identification. Inclusion criteria were as follows: both adult and pediatric patients had to have a thoracolumbar fracture that was treated surgically on the initial presentation, a documented neurological examination at the initial encounter and follow-ups, and a minimum of six months of follow-up. Radiographic information that confirmed the diagnosis of a traumatic fracture and follow-up imaging demonstrating the integrity of the instrumentation and rate of fusion was required. Exclusion criteria included nonoperative management, no documentation of a detailed neurological assessment upon presentation or insufficient postoperative follow-up, or if sufficient information was not provided about their status postoperatively and if they had insufficient follow-up or were not seen on follow-up at all after hospital discharge following the initial surgery. To ensure the quality of the study design, the Strengthening the Reporting of Cohort Studies in Surgery guidelines were followed [9].

Measured variables

Basic patient demographics were recorded, including age, sex, mechanism of injury, and past medical history. The radiographic level(s) of injury defining the fracture involved and all other associated spinal injuries were recorded. A baseline neurological status was also recorded, including the American Spinal Injury Association (ASIA) score [10], the Frankel score [11], and the ability to ambulate independently with or without an assistive device. If multiple fractures were evident, the level requiring surgery was labeled as the injury site. Intraoperative variables included surgical approach selection, construct length by the level of vertebral bodies fused, presence of cerebrospinal fluid (CSF) leak, and total estimated blood loss (EBL) in milliliters. All patients underwent anterior corpectomy followed by posterior stabilization or vice versa. Thus, all surgeries were a two-stage intervention at a minimum. Postoperative examinations were reported in terms of both the ASIA scale and Frankel score for preoperative comparison. Additional postoperative variables emphasized the patient's overall length of stay (LOS), and postoperative complications such as the presence of new urinary tract infection, surgical site infection (SSI), venous thromboembolisms (either pulmonary emboli or deep venous thrombosis (PE/DVT)), and new neurological deficits.

After discharge from the hospital, all patients were required to have a minimum of one follow-up between four and six months. Follow-up was divided into three different durations. Short-term follow-up occurred between zero and six months. Intermediate follow-up was between six and 18 months and long-term follow-up was greater than 18 months. Radiographic evaluation of hardware integrity and extent of osseous fusion was performed using CT or X-ray at each follow-up. Serial ASIA scales and Frankel scores were assessed in addition to the persistence or resolution of preoperative neurological deficits to determine the extent of neurological improvement and functional outcome. If there was no postoperative documentation confirming continued deficits, issues such as urinary retention were deemed resolved.

Literature search and review

To ensure a comprehensive literature review, we conducted a search of the PubMed database using the single key phrase “fusion for thoracolumbar traumatic fractures,” with a limit to articles published in English within the past five years that examined human subjects. Articles of interest addressed the initial surgical fixation of thoracolumbar fractures at the time of injury and contained sufficient demographic data, including the number of levels fused, EBL, alignment (as indicated by the rate of subsidence or development of kyphotic angulation), rate of revision surgery, complications, and neurological status before and after surgery, which would indicate a change resulting from the surgery or a lack of an expected improvement. Studies missing only one data point but still deemed relevant were also included. Exclusion criteria included case reports and papers that did not include or discuss the surgical treatment of thoracolumbar fractures or contain sufficient demographic information for comparison to our study. Articles deemed appropriate for inclusion were organized using the Preferred Reporting Items for Systematic Reviews and Meta-Analyses (PRISMA) flowchart of meta-analysis [12]. Titles and abstracts were reviewed, followed by an assessment of the full-length articles for inclusion.

Statistical analysis

A statistical analysis of the data collected from the patient charts was conducted using Microsoft Excel (Microsoft Corporation, Redmond, WA). Due to the lack of individual patient data in the literature, direct statistical comparisons of averages between our patient cohort and the control group were not possible. Therefore, all demographic, preoperative, intraoperative, and postoperative variables were tabulated and reported as both averages and percentages and compared directly.

## Results

Demographics

A total of 40 patients met the inclusion criteria. The average age was 41.8 years with a total of 17 females and 23 males. The most common mechanism of injury was fall (n = 31), followed by motor vehicle collision (n = 6) and other causes (n = 3). No patients had any significant past medical history that pertained to the original injury or any implications for the surgical fixation of the fracture. In terms of preoperative neurological status, the majority of patients were intact (ASIA E, n = 21), or had minimal extremity weakness (ASIA D, n = 11). A minority of patients had significant extremity weakness earning a score of ASIA C (n = 4), and one patient had a score of ASIA A. It is important to note that for three patients, due to concomitant orthopedic injury or altered mental status, a full 24 hours was not given to obtain a full ASIA scale, but a baseline neuromotor exam was able to be established. In terms of bladder function, 35 had preserved function while five were impaired. A total of 26 patients could ambulate preoperatively while 14 patients could not ambulate due to a spinal cord injury, other orthopedic injuries, or altered mental status, which prohibited participation. In terms of the radiographic level of injury, L1 and T12 were most commonly involved (n = 20 and n = 6, respectively). One patient did have kyphoplasty prior to surgery and failed this treatment. A summary of baseline patient demographics is summarized in Table 1.

**Table 1:** Baseline demographics and preoperative status ASIA: American Spinal Injury Association; MVC: motor vehicle collision.

Variable	Value
Number of patients	40
Average age	41.8
Females	17
Males	23
Mechanism of injury	
Fall	31
MVC	6
Other	3
Exam on presentation (ASIA score)	
A	1
B	0
C	4
D	11
E	21
Unknown	3
Bladder dysfunction	
Yes	5
No	35
Ability to ambulate	
Yes	26
No	14
Level of injury	
T8	1
T11	1
T12	6
L1	20
L2	6
L3	4
L4	1
L5	1

Surgical and intraoperative data

The majority of patients underwent a two-stage anterior corpectomy followed by a bi-segmental posterior fusion, or vice versa, except for one patient who required additional fixation and was fused two levels above and below the injury. The average blood loss was 670 milliliters cumulatively. No CSF leaks occurred. Most patients underwent each stage on a different day, requiring two days of surgery (n = 37), with exceptions for two patients who underwent both stages in one day, and one patient who required three separate stages. There were no instances of intraoperative neuromonitoring signal loss. Two SSIs occurred postoperatively, one involving the posterior incision and the other involving the corpectomy access site. One patient with the latter infection required revision surgery after a prolonged LOS of 28 days, while the other was successfully treated with antibiotics. A general surgeon performed surgical exposure for the corpectomy. One patient who incurred an EBL of 700 milliliters, experienced hypovolemic shock postoperatively. The majority of patients were discharged home (n = 21), while the rest were discharged to an acute rehab facility (n = 14). A summary of operative data points is provided in Table 2.

**Table 2:** Operative characteristics and hospital stay LOS: length of stay; PE/DVT: pulmonary emboli/deep venous thrombosis.

Variable	Value
Number of levels fused	3 (except for 1 patient)
Average total blood loss (cortectomy and posterior fusion)	670 cc
Incidence of CSF leak	None
Number of stages	
1	2
2	37
3	1
Average LOS	11.7 days
Postoperative complications	
PE/DVT	2
Infection	2
Hypovolemic shock	1
Disposition	
Home	21
Acute rehab	14
Skilled nursing facility	1
Other	4
Instrumentation	
Fusion rate	97.5%
Hardware failure	1 (subsidence)
Revisions	0

Postoperative outcomes

During the first period of follow-up, only one patient was re-admitted for SSI. The second patient with an SSI had a LOS of 28 days. She returned to the OR and was re-operated during her original hospitalization. X-rays and CT scans obtained at follow-up demonstrated a 97.5% fusion rate. Preserved alignment was appreciated in all cases except for the single incident of kyphosis, which was also complicated by hardware subsidence into the adjacent vertebral body. Here, there was focal kyphosis of 18.6 degrees over the fusion, yet due to adequate boney fusion and a lack of related symptoms, no revision surgery was needed. A total of 33 patients were ambulatory within the first six months, and 35 reported having normal bladder function. ASIA score of E was the most common sensorimotor outcome and occurred in 32 patients, and a Frankel score of E was seen in 31 patients. Only 65% of patients (n = 26) were seen at the follow-up between six and 18 months postoperatively. At this time, the majority of patients had an ASIA score of E, and a Frankel score of E (n = 25 and n = 24, respectively). As of 18 months of follow-up, only 32.5% of patients were able to be assessed (n = 13). At this time, all patients were ambulating independently with or without an assistive device and had preserved bladder function. An ASIA score of E and a Frankel score of E were observed in 12 and 11 patients, respectively. No patients had any long-standing inability to ambulate or retain urine. A summary of follow-up and neurological assessments is provided in Table 3.

**Table 3:** Postoperative follow-up and neurological function ASIA: American Spinal Injury Association.

	6-month follow-up	6 to 18-month follow-up	>18-month follow-up
# (%) Patient follow-up	40 (100)	26 (65)	13 (32.5)
Ability to ambulate			
Yes	33	25	13
No	7	1	0
Bladder function			
Yes	35	25	13
No	5	1	0
ASIA score			
A	1	0	0
B	0	0	0
C	3	0	0
D	5	1	1
E	31	25	12
Frankel score			
A	1	0	0
B	0	0	0
C	3	0	0
D	5	2	2
E	31	24	11

## Discussion

The literature was searched using the criteria delineated above, which identified six studies [13-18]. These studies, summarized in Table 4, included a mix of dual-armed prospective studies, single-arm prospective studies, and retrospective reviews. Most studies evaluated single-level corpectomies or bi-segmental posterior fusion. Some compared minimally invasive surgery (MIS) to open surgery. The recorded blood loss varied widely, with some patients losing over 2 liters of blood and requiring a transfusion. Fusion rates were high in all studies, ranging from 84.2% to 100%. Postoperative alignment studies that examined posterior-only constructs had larger degrees of kyphosis compared to those that involved anterior cage placement followed by posterior fusion. Studies that examined corpectomies with cage placement had small rates of subsidence and preserved alignment. All studies reported excellent neurological outcomes, although some studies reported no improvement in patient symptoms [16,17], and one study reported a few incidents of neurological deterioration [13]. The complication rate ranged between 2.6% and 15.3%, while the overall revision rates were low for all studies, generally involving only one patient.

**Table 4:** Literature review EBL: estimated blood loss; MIS: minimally invasive surgery.

Study	Type of study	# of levels fused	EBL	Fusion rate	Alignment	Neurological function change as a result of surgery	# of patients with complications	Revision rate (# of patients)
Podet et al. (2020) [13]	Comparison of MIS and open	89.8% had single-level corpectomy, 18.5% had multi-level corpectomy	2211 ml vs. 2406 ml	90.6% vs. 100%	2 (6.3%) had subsidence, no reported degrees	2 (3.4%) experience neurological deterioration	9 (15.3%)	1 patient, 1.7%
Smits et al. (2018) [14]	Retrospective review	Anterior: 39% were 1 level, 59% were 2 levels, and 2% were 3 levels	600 ml	98%	6.8 degrees of correction loss over time	Only included intact patients, and there was no reported change in function	5 (10%)	0%
Wang et al. (2022) [15]	Comparison of Wiltse approach to transforaminal interbody	3 levels, fractures level, 1 above and below	437.84 vs. 862.7 ml	86.8% vs. 84.2%	Improvement with surgery but no change after	No deterioration and both had improvement in Frankel score	2 total infections (2.6%), 3 CSF leaks (3.9%)	2 for infection, none for instrumentation failure
Brembilla et al. (2022) [16]	Prospective single-arm study	18 had 3 levels, 1 had 4 levels, 1 had 5 levels	294.5 ml	94.4%	7.57 degrees of kyphosis at the final follow up	4 patients had no improvement, none were worse after surgery	0 intraoperative complications, no other reported event	1 (5%) had implant failure
Lang et al. (2021) [17]	Retrospective study	81.3% were bi-segmental, 18.8% had longer (up to 4 segments)	Not recorded	97.9%	2.4 degrees at last follow	4 continued to have complete deficits, 3 showed no improvement	10.4% overall rate, 4.2% infection	4.2%
Scholz et al. (2018) [18]	Prospective, randomized trial: anterior and posterior to just posterior fusion	3 levels posteriorly for all	Not recorded, no patient needed transfusion	The rate was determined radiographically but not reported	8.3 degrees for anterior-posterior vs. 15.6 degrees for posterior	Only examined intact patients, no postoperative deficits	1 delayed wound healing, 1 loss of correction	1 (4.7%)

In this study, we present our institutional experience for the surgical treatment of thoracolumbar burst and chance fractures via a combined anterior/posterior approach to achieve a 360-degree surgical fixation. Our patient population was mostly young males who sustained an injury from a fall. Prior to surgery, patients had a good neurological exam with minimal deficits. The anterior corpectomy followed by a posterior fixation permitted very high fusion rates (97.5%). This method allowed for a shorter vertical segment of fusion, which preserved mobility despite the injury occurring at a transitional level in the thoracolumbar junction. All patients had either preservation of neurological function or improvement in preoperative deficits. Complication rates were low, and the majority of patients were discharged home without the need for revision surgery for hardware-related complications. No revision surgery for hardware-related complications was needed. Because of these results, we feel that circumferential fusion involving both the anterior and posterior anatomic compartments increases the strength of the construct, necessitating a less extensive fixation and thus preserving additional motion segments as compared to larger posterior-only constructs.

There is ongoing debate regarding the optimal construct for surgical stabilization of thoracolumbar fractures, with particular uncertainty surrounding the number of levels that should be fused [19]. This is further complicated by the fact that not all fractures are equivalent, and some are better suited for short-segment constructs, such as flexion-distraction injuries, mild burst fractures, or fracture/dislocation injuries [20]. The McCormick score is a useful tool, giving guidance as to which fractures require sole posterior fixation (a score <6 being the cutoff) [21]. Additional index screws can increase construct strength, but short-segment constructs are still generally inferior to long-segment constructs [22]. Intermediate screw placement has been associated with improved radiographic outcomes and a reduced incidence of implant failure [23]. Additionally, selective placement of screws at the fracture level can aid in alignment and increase construct strength [24]. When considering the location of screw placement itself, cortical screws have been proven equivalent to pedicle screws in terms of fixation strength [25]. However, bone quality and other individual patient factors should be considered when evaluating the choice of screw trajectory. With respect to bone quality, the density as approximated in Hounsfield units on CT can often act as a surrogate indicator of bone health preoperatively [26]. In cases where the construct fails biomechanically, more extensive constructs with high-grade osteotomies are often necessary to achieve fixation [27].

The chief biomechanical property of our construct design is its high strength. We provide significant anterior column support and fixation, resulting in a high fusion rate, which is superior to an isolated posterior construct in terms of strength. Our surgical approach has a relatively low EBL and low complication rate and preserves or improves neurological function. All of our statistics are comparable or superior to that reported in the literature across all measured values, with only one patient experiencing subsidence, achieving full fusion, and not requiring revision. All other patients had complete alignment preservation, as we had achieved a high degree of alignment preservation not seen in the literature with alternative construct designs. Khare et al. reported an improvement in vertebral height of only 78% with a decrease in kyphotic Cobb angle to 27.8 degrees when an isolated posterior short segment fusion was employed [28]. Choovongkomol et al. showed that kyphotic progression can be seen in as much as 35.2% of patients who undergo isolated short-segment posterior fixation when either the posterior ligamentous complex was injured or when intermediate screws were used [29]. Percutaneous fusion represents an alternative surgical approach that may be associated with better correction [30]. Fortunately, patients who undergo surgery in a delayed fashion can still have an equal reduction, as there is no relationship between delayed surgery and the extent of reduction [31]. Given the lack of consensus about the most effective approach biomechanically, we posit that our approach is superior based on our outcomes and relatively increased rates of preserved alignment.

Our approach is driven by the consideration that a larger relative surface area for fusion increases the likelihood of successful fusion and reduces the risk of construct failure. The anterior column support promotes the preservation of thoracolumbar alignment, which can decrease the rate of adjacent segment disease. The increased fusion rates indicated in our study also decrease the risk of developing kyphotic deformity often associated with isolated posterior approaches. Consequently, more extensive corrective surgery was not necessary.

We believe that circumferential decompression via corpectomy and posterior decompression allows for complete neural element decompression, enabling patients to achieve maximal neurological recovery. For our study, this approach is associated with shorter hospital stays and a higher likelihood of discharge to home. Moreover, a shorter construct preserves motion segments, which is particularly significant for pediatric patients in promoting a return to normal activity. While long-term follow-up is necessary to confirm our hypothesis, we believe that our overall shorter segmental fusion, which enhances mobility, will be linked with a decreased rate of adjacent segment disease and subsequent adjacent level failure in the future. One hypothetical reason for this is that a tremendous amount of strain is transmitted from the screw into the rod in a modular base system in cases of isolated posterior fixation. Theoretically, as a result, as vertical forces are placed on the construct, the maximal force is placed on the construct and not in places that promote fusion. Over time, the force of gravity increases the risk of kyphosis and subsequent construct breakdown, leading to implant failure. Conversely, a cage that potentially provides anterior and middle column support allows for direct transmission of gravitational force from the cephalad vertebral body through the implants and into the caudal vertebral body. This increased pressure is not only transmitted more effectively but also promotes more effective fusion as described by Wolff’s law. This is our hypothesis. This simultaneous preservation of alignment with increased fusion rates observed with this type of construct explains the effectiveness of the approach. We demonstrate that for burst and chance type fractures with significant loss of height or compression of neural elements, corpectomy followed by posterior fixation is a superior approach in the right patient population, as it increases rates of fusion with the maintenance of alignment and good neurological function. Also, we suspect that maximal motion segment preservation through the usage of the shortest and strongest construct will ultimately decrease the rate of construct failure and adjacent segment disease, particularly in the young population affected by spine trauma.

Limitations of this study are predominantly inherent to its retrospective methodology for both the analysis of our institutional cohort and the literature review and include selection bias, confounding variables, and lack of a comparative control group. The relatively small sample size and heterogeneous composition of the population further challenge the generalizability of our results to the greater patient population. Finally, a long-term follow-up beyond 18 months is necessary to confirm whether the increased mobility of the thoracolumbar spine associated with limited fusion decreases the incidence of adjacent segment disease. Exploring this would warrant further research.

## Conclusions

Thoracolumbar fractures remain a common pathology that requires neurosurgical intervention. We present data that 360-degree fusion via a combined anterior/posterior approach yields a high degree of fusion and thus enables a relatively short construct while simultaneously achieving stability. Our patient cohort was young, predominantly male, intact neurologically, and experienced falls as the most common etiology. Postoperatively, all patients had preserved neurological function and experienced low blood loss and few complications. We believe that our approach is superior to others reported in the literature due to its high fusion rate and effectiveness in preserving alignment and mobility. Our data support the notion that this approach is particularly effective at the thoracolumbar junction where preservation of motion has significant clinical implications. While future prospective studies are needed to validate our findings, we believe this surgical approach represents the ideal surgical intervention for correcting thoracolumbar bursts and chance fractures.
